# Machine Learning to Study Social Interaction Difficulties in ASD

**DOI:** 10.3389/frobt.2019.00132

**Published:** 2019-11-29

**Authors:** Alexandra Livia Georgescu, Jana Christina Koehler, Johanna Weiske, Kai Vogeley, Nikolaos Koutsouleris, Christine Falter-Wagner

**Affiliations:** ^1^Department of Psychology, Institute of Psychiatry, Psychology and Neuroscience, King's College London, London, United Kingdom; ^2^Department of Psychiatry and Psychotherapy, University Hospital of Cologne, Cologne, Germany; ^3^Department of Psychiatry and Psychotherapy, Medical Faculty, LMU Munich, Munich, Germany; ^4^Institute of Neuroscience and Medicine, Cognitive Neuroscience (INM-3), Research Center Juelich, Jülich, Germany; ^5^Institute of Medical Psychology, Medical Faculty, LMU Munich, Munich, Germany

**Keywords:** autism spectrum disorder, machine learning, nonverbal synchrony, support vector machine, motion energy analysis, classification, intrapersonal synchrony, nested cross-validation

## Abstract

Autism Spectrum Disorder (ASD) is a spectrum of neurodevelopmental conditions characterized by difficulties in social communication and social interaction as well as repetitive behaviors and restricted interests. Prevalence rates have been rising, and existing diagnostic methods are both extremely time and labor consuming. There is an urgent need for more economic and objective automatized diagnostic tools that are independent of language and experience of the diagnostician and that can help deal with the complexity of the autistic phenotype. Technological advancements in machine learning are offering a potential solution, and several studies have employed computational approaches to classify ASD based on phenomenological, behavioral or neuroimaging data. Despite of being at the core of ASD diagnosis and having the potential to be used as a behavioral marker for machine learning algorithms, only recently have movement parameters been used as features in machine learning classification approaches. In a proof-of-principle analysis of data from a social interaction study we trained a classification algorithm on intrapersonal synchrony as an automatically and objectively measured phenotypic feature from 29 autistic and 29 typically developed individuals to differentiate those individuals with ASD from those without ASD. Parameters included nonverbal motion energy values from 116 videos of social interactions. As opposed to previous studies to date, our classification approach has been applied to non-verbal behavior objectively captured during naturalistic and complex interactions with a real human interaction partner assuring high external validity. A machine learning approach lends itself particularly for capturing heterogeneous and complex behavior in real social interactions and will be essential in developing automatized and objective classification methods in ASD.

## Introduction

Autism spectrum disorder (ASD) is an umbrella term for neurodevelopmental conditions characterized by severe difficulties in social interaction and communication, as well as by repetitive behaviors and restricted interests (American Psychiatric Association, 2013). The prevalence rates of ASD are on the rise (Elsabbagh et al., 2012) and diagnostic services are experiencing an increased demand, in particular in adults seeking diagnostic advice (Murphy et al., 2011). Diagnostics according to medical guidelines are time-consuming, the clinical assessment is complicated by the phenotypic heterogeneity and the language-dependency of assessment with verbal skills being affected by the ASD.

Recently, computational methods of classification have been employed to increase diagnostic reliability and efficiency (Thabtah, 2018). In particular, machine learning (ML) employs algorithms to uncover patterns in complex datasets, which are utilized to improve decision making. ASD diagnostics come down to a decision-making problem that can be supported by automated models (classifiers) using ML to decide whether a newly assessed patient has ASD or not. This works by splitting available data into a training set, on which an algorithm is trained, which is then applied to a test set, resulting in a measure of accuracy of the resulting model. Without making assumptions ML finds classification solutions in a data-driven, bottom-up approach that can be applied to individual prediction making (Dwyer et al., 2018). The primary purposes of using ML are (1) to reduce assessment time to reach a diagnostic decision in order to provide quicker access to health care services, (2) to improve diagnostic reliability, and (3) diagnostic validity by reducing dimensionality of input data so as to identify those features that have the most diagnostic value in ASD (Thabtah, 2018). However, first applications of ML in studies on autism diagnostics have been inconsistent in terms of methodology and outcome, with inconsistent classification accuracy and specificity.

The aim of the present paper is twofold: First, we aim to give an overview of previous research that has attempted to apply ML methods to the classification of ASD, while suggesting guidelines for future research in terms of setup and algorithm design. Second, in a proof-of-principle analysis of data from a social interaction study we aim to establish the potential of using full-body non-verbal behavior data extracted from video recordings of naturalistic social interactions to classify autistic adults.

## Machine Learning Applications in the Classification of ASD

First ML attempts in ASD have been used with the aim of shortening ADOS [Autism Diagnostic Observation Schedule, (Lord et al., 2000)] and ADI-R [Autism Diagnostic Interview, (Lord et al., 1994)] administration time by item-reduction yielding a classification accuracy of autism vs. typically-developing (TD) individuals of up to 99.9% (Wall et al., 2012a,b; Bone et al., 2016). In a similar attempt to predict case status words and expressions contained in 8 year old children's developmental evaluations across a network of multiple clinical sites were used for algorithm development (Maenner et al., 2016) with 86.5% prediction accuracy and high concordance with the respective clinician. Home videos of children have been rated by naïve and/or expert raters in terms of ASD-typical behavior and ratings fed into a predictive model along with other features of the diagnostic process (Glover et al., 2018; Tariq et al., 2018). However, while all these first studies using ML in ASD yield fairly high accuracies, the features utilized for classification are still highly subjective and not independent of the respective clinician who bases the diagnostic decision on just those features (circularity). Importantly, when using subjectively influenced data, resulting classification algorithms must be validated in an independent sample in order to prevent circularity.

An increasing number of studies are also using ML to separate individuals with ASD from TD individuals based on neuroimaging data. For example, Ecker et al. (2010) used regional gray and white matter volume measures from whole-brain structural MRI scans of individuals with ASD to investigate their diagnostic value. They used a common variant of ML, the support vector machine (SVM). This is an algorithm aiming at finding a boundary (the so-called “hyperplane”) that can be used to optimally classify groups while being able to generalize to new cases (Dwyer et al., 2018). In their sample, the SVM correctly classified individuals with ASD and controls on the basis of their neuroanatomy with about 80% accuracy (Ecker et al., 2010). These original observations are supported by findings from several other neuroimaging studies with similar levels of classification accuracy in younger age groups (Wee et al., 2014), females with ASD (Calderoni et al., 2012) and with various anatomical and functional measurements (Coutanche et al., 2011). These results based on objective data are very promising, although not widely applicable due to high costs.

## Whole-Body Movements as a Feature in ML Algorithms in ASD

Another source of objective data with high potential for diagnostics can be found in the motor domain. Approximately 80% of children with ASD are suspected to exhibit pronounced motor difficulties (Green et al., 2009). Difficulties with balance, gait, movement speed and timed movements have demonstrated to hold a high level of discrimination between children with ASD and TD children (Jansiewicz et al., 2006) and correlate strongly with measures of social and communicative functioning (Parma and de Marchena, 2016). Hence, movement parameters of social interactions in ASD should be investigated for their potential as a diagnostic marker.

Particularly relevant for ASD motor symptomology are gestures and non-verbal communicative behaviors (Georgescu et al., 2014). Accordingly, atypical non-verbal behavior has been included in the DSM-5 criteria for ASD. Yet, the assessment is not straightforward or standardized so far and is hampered by the fact that non-verbal behavior is not necessarily reduced in ASD, but abnormal in the *quality* of its temporal coordination with own verbal output (de Marchena and Eigsti, 2010) and that of an interaction partner. Literature provides evidence for aberrations in temporal processing (Allman and Falter, 2015) and time experience in ASD (Vogel et al., 2019), potentially affecting non-verbal communication. In fact, findings have shown that ASD can be characterized by increased temporal resolution associated with the severity of (non-verbal) communication impairments in ASD (Falter et al., 2012, 2013; Menassa et al., 2018; but see Isaksson et al., 2018).

Recently, movement in ASD has taken up increasing interest (for a review see Bo et al., 2016). In a proof-of-concept study to explore whether low-functioning children with ASD could be identified by means of a kinematic analysis of a simple motor task, 15 children with ASD and 15 TD children (2–4 years) were asked to pick up a ball and drop it into a hole while their movements were recorded using a motion tracker (Crippa et al., 2015). Seventeen kinematic parameters were extracted from the upper-limb movement and seven of these were found significant for discrimination. The classifier distinguished ASD from non-ASD with a classification accuracy of 96.7%, suggesting the validity of assuming a motor signature of ASD. Reach and throw movements of 10 ASD and 10 TD children were analyzed for “peculiar features” using ML and fed into a classification algorithm yielding an accuracy of 92.5% (Perego et al., 2009). Furthermore, Li et al. (2017) extracted 40 kinematic parameters of imitative movements and identified 9 of them that best describe variance of participant groups, resulting in a classification accuracy of 93%.

These studies demonstrate the potential of using kinematic biomarkers in diagnostics of ASD. However, the movements under investigation were staged, thus, highly unnatural. Yet, it has been established that individuals with ASD have particular difficulties with spontaneous “on-line” social interaction requiring intuitive decisions and behavior (Redcay et al., 2013) constituting an urgent need to move this type of research to more external validity and investigate movement in a more naturalistic context.

## Classification Using Intrapersonal Synchrony: A Proof-of-Concept Study

**Figure 1:**
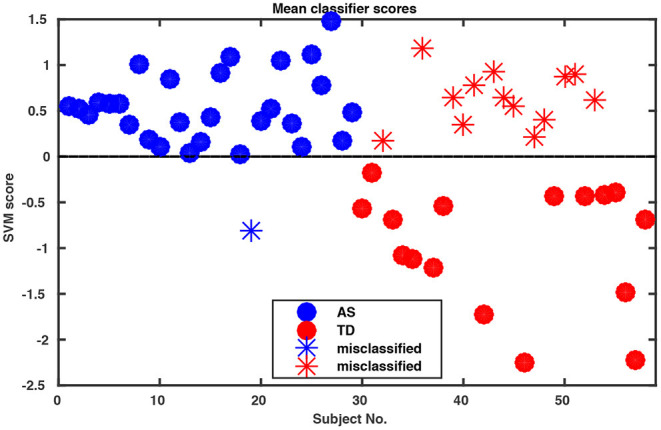
Decision scores of SVM classification performance. The algorithm assigns a score to each participant indicating the probability of this participant as belonging to Group 1 or 2 (in our case ASD vs. TD) where the decision boundary between the two groups is zero. Notably, our algorithm misclassified only one of the ASD participants.

Whole-body movements in more naturalistic conversations were tested for their classification potential in 29 high functioning adults with ASD and 29 TD individuals. The data for this investigation came from a study on interpersonal coordination in dyadic interactions (Georgescu et al., under revision). The autistic participants were diagnosed and recruited at the Autism Outpatient Clinic of the Department of Psychiatry, University Hospital Cologne, Germany. The sample included only patients with the diagnoses high-functioning autism (ICD-10: F84.0) or Asperger syndrome (ICD-10: F84.5). Two medical specialists confirmed the diagnosis independently in clinical interviews, according to the criteria of the International Classification of Diseases (ICD-10) and supplemented by extensive neuropsychological examination. The TD sample was recruited online from the student and staff population at the University of Cologne and the University Hospital of Cologne, Germany. The study was conducted with the approval of the local ethics committee of the Medical Faculty of the University of Cologne. Participants were paired to conduct five 5 min social interaction tasks. Conversational dyads consisted of either two TD individuals, two individuals with ASD or a TD individual with an individual with ASD. An ice-breaker task, two debating tasks, a meal-planning task and a roleplay were included resulting in a total of 145 videos of social interactions (for more information, see Georgescu et al., under revision). All conversations were recorded in a room with standardized artificial lighting and using a high-definition video camera (Panasonic DV C Pro HD P2), mounted on a tripod 320 cm away from the chairs which were 60 cm apart from each other. Since one of the MIXED dyads did not understand instructions on the ice-breaker task, for the purpose of this analysis the whole task was abandoned, resulting in a total of 116 videos submitted for final analysis. Intrapersonal Synchrony between the head and upper body was quantified using Motion Energy Analysis, a widely used semi-automated frame-differencing method that continuously monitors the amount of movement occurring in manually pre-defined regions of interest and the method of lagged cross-correlations (Nagaoka and Komori, 2008; MEA; Altmann, 2011; Ramseyer and Tschacher, 2011). MEA offers the advantage of a constraint-free, objective analysis tool for non-verbal behavior (e.g., Ramseyer and Tschacher, 2011; Schmidt et al., 2012; Paxton and Dale, 2013). This method has been used to capture body movement in different contexts (e.g., Grammer et al., 1999; Ramseyer and Tschacher, 2011, 2014; Schmidt et al., 2012, 2014; Paxton and Dale, 2013). MEA and other frame-differencing methods have been successfully used in clinical research before (e.g., Kupper et al., 2015) and in particular in autism (Noel et al., 2017; Romero et al., 2017, 2018). We followed the MEA pipeline described in Ramseyer and Tschacher (2014). We manually selected two regions of interest (ROI) for each participant, covering (1) the head and (2) the rest of the body including the legs. Changes in grayscale values in these ROIs were detected and separately recorded as two continuous time series measuring the amount of movement in the head and the body region of each person. Data were submitted for quantification of Intrapersonal Synchrony (for more information on the MEA procedure in general, please see Ramseyer and Tschacher, 2014 and on this sample, Georgescu et al., under revision). Input time series were smoothed and scaled to account for different-sized ROIs using custom software in R (package rMEA, Kleinbub and Ramseyer, 2019) and cross-correlated in windows of 60 s with a time lag of ±5 s (step size 0.04 s). Windows were not allowed to overlap. The resulting 1,004 lagged cross-correlations were then z-standardized and aggregated over the four conditions for every participant, yielding 4,016 features per participant which were implemented in the open-source machine learning tool NeuroMiner (https://www.pronia.eu/neurominer/). A support vector machine with linear kernel was chosen as a classification algorithm, a multivariate supervised learning technique widely used in psychiatric research (Bone et al., 2016; Duda et al., 2016). Our repeated nested *k*-fold cross-validation (CV) structure consisted of 10-folds and five permutations for the outer cross-validation cycle (CV_2_) and repeated 5-by-5-fold inner cross-validation cycle (CV_1_), with participants being shuffled prior to each definition of folds. This way, the data available for training was maximized while ensuring enough heterogeneity within the inner test sample to avoid overfitting and create stable models. Parameter optimization was performed in CV_1_, while model performance was evaluated in CV_2_. Prior to analysis, data was preprocessed using principal component analysis (PCA) for dimensionality reduction, retaining the principal components that cumulatively explained 80% of the variance in each CV_1_ fold, and subsequently, scaled feature-wise from 0 to 1. The slack parameter C was estimated in the inner CV cycle using eight parameters ranging from 0.015625 to 16. Overall classification performance resulted in 75.9% accuracy (Table 1). Remarkably, sensitivity was 96.6%, correctly classifying all but one individual with ASD (Figure 1).

**Table 1:** Performance metrics of the ASD vs. TD SVM classifier.

True positives/true negatives	28/16
False positives/false negatives	13/1
Accuracy [%]	75.9
Sensitivity [%]	96.6
Specificity [%]	55.2
Area under the curve	0.71

*For detailed explanation of performance metrics please refer to Dwyer et al. (2018)*.

Thus, with a portable and inexpensive video-setup in a naturalistic setting and a semi-automated analysis pipeline, we reached a good diagnostic classification of ASD within four 5 min interaction excerpts on the mere basis of objective motion data. Feeding further clinical and interaction variables into the algorithm promises a high potential for classification (see Future Perspectives section).

## Methodological Issues in Machine Learning Approaches to Classifying ASD

Unlike e.g., Bone et al. (2016) or Li et al. (2017), most ML studies in ASD research have relied on simple cross-validation (CV) methods. This increases the likelihood of choosing an overly optimistic model (Cawley and Talbot, 2010). We therefore suggest the application of a second layer of CV to allow for parameter selection and model performance evaluation to not be performed on the same data and to prevent overfitting. The test fold is completely held out until parameter optimization within the inner CV cycle is achieved by splitting the training data once more into an (inner) test and (inner) training set. The optimized models can then be tested for generalizability on the outer test fold. This so-called nested CV maximizes generalizability and has now been established as a gold standard procedure in psychiatric research (Dwyer et al., 2018). In order to account for the small sample sizes in ASD research, often predictions are made in a leave-one-out approach whereby only one individual's data is held out in the test set while parameters are optimized on the others (Crippa et al., 2015; Li et al., 2017). Especially, for ASD with its highly heterogeneous phenotype, leave-one-out creates overly variable test sets, rendering model outcomes unstable (Varoquaux et al., 2017). This can be prevented through k-fold nested CV and simultaneous permutation of individual data sets within the inner cross-validation cycle (Dwyer et al., 2018). An overview of best-practice standards is outlined below.

## Future Perspectives

Impairments of non-verbal communication are seen across the entire spectrum of ASD warranting the use as a behavioral biomarker. Yet, its intricacy requires multivariate analysis methods to capture complex interdependencies across domains. Machine learning offers the potential to incorporate high-dimensional data for the detection of underlying mechanisms and classification if certain minimum practice requirements are fulfilled (see Box 1).

Box 1 **Minimum requirements for reliable clinical application of ML in ASD research (adapted from Dwyer et al., 2018)**

- Combination of objective variables and standard diagnostic measures as input features to classify ASD.
- Use of nested CV as a standard procedure.
- Prevent unstable model outcomes through *k*-fold CV.

In our proof-of-principle study, we were able to classify high-functioning adults with ASD from TD adults on the mere basis of non-verbal intrapersonal motion synchrony in social interactions with an accuracy of 75.9%, which can be regarded a conservative estimate on the basis of a state-of-the art ML approach. Due to relatively small sample sizes available with high phenomenological heterogeneity in ASD, it is of utmost importance to choose adequate methods of cross-validation in order to maximize generalizability. The use of repeated nested cross-validation prevents overfitting and should be incorporated as a standard procedure in ML applications. However, given our rather limited sample size, the next steps for future research will be to apply the resulting algorithm to a completely new and larger data set and to investigate its transdiagnostic specificity across different psychiatric disturbances.

Future research should furthermore consider combining multiple non-verbal communication parameters and clinical data (e.g., questionnaires) in order to improve prediction and classification accuracy further and to possibly detect potential associations across domains. For instance, peculiarities in eye-gaze (Merin et al., 2007; Georgescu et al., 2013) and facial expression (McIntosh et al., 2006) in ASD demonstrate feasible approaches.

One future avenue would be to explore methods to quantify non-verbal behavior in a fully-automated fashion. In the present proof-of-principle study, a dataset was used that was analyzed using MEA, a classic frame-differencing approach. It has been shown that MEA is able to capture movements and even complex coordinative patterns to a similar extent as more expensive motion capture equipment such as the Polhemus (Romero et al., 2017). A main advantage for autism research of this method of extracting whole-body motor movement is that it does not involve any wearable technology. Given the hypersensitivity exhibited by many individuals with ASD, not having to add any attachable piece of equipment or body suit to their bodies is helpful. However, while MEA automatically detects pixel changes, corresponding regions of interest are drawn in manually. Although resulting values are standardized, there remains a subjective component. Computer vision tools that can estimate the coordinates of limb positions and even extract gaze location and body poses would offer similar benefits while balancing out subjective biases in the motion extraction process (Marín-Jiménez et al., 2014; Mehta et al., 2017; Tome et al., 2017; Cao et al., 2018). In addition, they offer even more flexibility, given it could be possible to include less strict and standardized experimental setups (no requirement for standardized camera or lighting conditions). However, the validity for movement extraction compared to other standard motion capture methods has not been demonstrated yet. Moreover, such tools vary greatly with respect to their susceptibility to tracking failures, or the type of videos they can support (single vs. multiple agent, indoor vs. outdoor etc.). Overall, with the current methodology that is available for motion extraction, the present semi-automated method offers a realistically applicable diagnostic value. Nevertheless, incredible advances are being made (Li et al., 2018; Tran et al., 2018) such that they are very promising tools for future non-verbal behavior in autism research and beyond.

Taken together, given the recent advances in predictive psychiatry, adequately applied ML offers the potential to fully capture the autistic phenotype in all its complexity with sufficient specificity across psychiatric disorders with a special focus on the spontaneous non-verbal behavior during social encounters with others and irrespective of clinician or site.

## Data Availability Statement

The video datasets generated and analysed during the current study are not publicly available due this being identifiable patient data from a sample that did not consent to their data being shared in any form.

## Ethics Statement

Written informed consent was obtained from all participants in accordance with the Declaration of Helsinki (1964). All participants received a monetary compensation for their participation of 50 Euros and were debriefed at the end. The study was conducted with the approval of the local ethics committee of the Medical Faculty of the University of Cologne.

## Author Contributions

AG, JK, and CF-W contributed equally to the drafting of this manuscript. AG provided the data. JW and JK performed the statistical analysis. All authors contributed to the manuscript revision, read, and approved the submitted version.

### Conflict of Interest

The authors declare that the research was conducted in the absence of any commercial or financial relationships that could be construed as a potential conflict of interest.
